# cccDNA-Targeted Drug Screen Reveals a Class of Antihistamines as Suppressors of HBV Genome Levels

**DOI:** 10.3390/biom13101438

**Published:** 2023-09-24

**Authors:** Ee Chee Ren, Nicole Ziyi Zhuo, Zhi Yi Goh, Isabelle Bonne, Benoît Malleret, Hui Ling Ko

**Affiliations:** 1 Singapore Immunology Network (SIgN), Agency for Science, Technology and Research (A*STAR), 8A Biomedical Grove, Immunos, #03-06, Singapore 138648, Singapore; nicole_zhuo@immunol.a-star.edu.sg (N.Z.Z.); goh_zhi_yi_from.tp@immunol.a-star.edu.sg (Z.Y.G.); benoit_malleret@nus.edu.sg (B.M.); 2 Immunology Translational Research Programme, Department of Microbiology & Immunology, Yong Loo Lin School of Medicine, National University of Singapore, 5 Science Drive 2, Block MD4, Level 3, Singapore 117545, Singapore; isabelle.bonne@nus.edu.sg; 3 Electron Microscopy Unit, Yong Loo Lin School of Medicine, National University of Singapore, MD1, Tahir Foundation Building, #B1-01, 12 Science Drive 2, Singapore 117549, Singapore; 4 Immunology Programme, Life Sciences Institute, Center for Life Sciences, National University of Singapore, #05-02, 28 Medical Drive, Singapore 117456, Singapore

**Keywords:** antihistamines, Bilastine, cccDNA, drug screen, hepatitis B antivirals, PARP inhibitors, PARP-1 antagonist, peptidomimetics, simplified fluorescence in situ hybridization, correlative light and electron microscopy

## Abstract

Chronic infection with hepatitis B virus (HBV) is incurable, as the current therapeutics cannot eliminate its persistent genomic material, cccDNA. Screening systems for cccDNA-targeting therapeutics are unavailable, as low copies of cccDNA in vitro complicate detection. To address this, cccDNA copies were massively increased to levels detectable via automated plate readers. This was achieved via continuous infection in a contact-free co-culture of an HBV generator (clone F881), which stably produced clinically relevant amounts of HBV, and HBV acceptors selected to carry high cccDNA loads. cccDNA-targeted therapeutics were then identified via reduced cccDNA-specific fluorescence, taking differences in the cell numbers and viability into account. Amongst the drugs tested, the H_1_ antihistamine Bilastine, HBVCP inhibitors and, surprisingly, current HBV therapeutics downregulated the cccDNA significantly, reflecting the assay’s accuracy and sensitivity in identifying drugs that induce subtle changes in cccDNA levels, which take years to manifest in vivo. Bilastine was the only therapeutic that did not reduce HBV production from F881, indicating it to be a novel direct suppressor of cccDNA levels. When further assessed, only the structurally similar antihistamines Pitolisant and Nizatidine suppressed cccDNA levels when other H_1_ antihistamines could not. Taken together, our rapid fluorescence cccDNA-targeted drug screen successfully identified a class of molecules with the potential to treat hepatitis B.

## 1. Introduction

Chronic infection with HBV cannot be easily cured, as its genomic material, cccDNA, resides in infected tissue reservoirs for years [1,2]. Current direct-acting therapeutics reduce the viral titer [3,4] but cannot eliminate cccDNA [5,6]. Nearly 300 million people worldwide remain infected with HBV [7,8], resulting in ~1 million deaths/year from liver-associated diseases. HBV reactivation has been reported for immunological and chemotherapies [9,10], which may, intriguingly, involve active replication in diseased extrahepatic tissues [11,12,13]. In fact, extrahepatic cancers are a leading cause of death in HBV carriers [14,15,16]. Therefore, to eradicate HBV and stamp out complications arising from reactivation, there is an urgent need to develop curative antivirals targeting cccDNA [4,17].

However, technology to detect cccDNA with high sensitivity and speed is underdeveloped, as cccDNA exists in low copies in vitro, necessitating time-consuming destructive processes and amplification [5,6]. cccDNA detection is further complicated by the inability to distinguish integrated viral replicons from de novo cccDNA in stable expression systems; thus, viral protein production from transcriptionally active cccDNA is often used as a proxy for the cccDNA titer [18]. As cccDNA reporter systems do not reflect true cccDNA levels from infection and are reliant only on the post-infection mechanism of nucleocapsid re-import to sustain nuclear cccDNA, such drug screen systems miss out on curative drugs that directly block cccDNA establishment upon infection [19,20].

To address the lack of a direct assay for cccDNA that is also sensitive to the blockade of HBV infection, we generated a cccDNA-targeting screening platform comprising an HBV generator engineered to continuously produce HBV, and HBV acceptor cells that accumulate high cccDNA loads. Transwell co-culture supports continuous infection while segregating the acceptor and generator, allowing the free passage of HBV and test drugs without contaminating cccDNA signals in acceptor cells with replicon signals from generator cells. The massively increased cccDNA load is detectable without amplification using molecular beacon probes that fluoresce only when linearized and bound to cccDNA, reducing the processing time in simplified fluorescence in situ hybridization (sFISH).

## 2. Materials and Methods

### 2.1. Cell Culture

Cell lines were purchased from ATCC and JCRB, grown at 37 °C in a humidified incubator with 5% CO_2_, and cultured in DMEM, EMEM or RPMI supplemented with 10% or 20% FCS, as recommended.

To generate F881, 4 × 10^5^ HuH-7 cells were seeded in a 6-well plate, co-transfected with 2.5 μg of *SNAI2*-targeting construct, 2.5 μg pTetOne HBV genotype B replicon and 250 ng linear hygromycin selection marker (Takara Bio Inc., Kusatsu, Shiga, Japan) using 5.5 μL Lipofectamine^TM^2000 in 500 μL OPTI-MEM. Transfected cells were treated with increasing minimally lethal doses of 250–350 μg/mL hygromycin 48 h post-transfection for 1 month, and surviving cells recovered in 20% FCS/DMEM for 1 week. Recovered cells were subjected to limiting dilution (0.5–64 cells/well) in 96-well plates and grown in 20% tetracycline-free FCS/DMEM supplemented with 30% conditioned media from HuH-7 for 3–4 weeks. The cells were visually inspected under microscope to identify single-cell clones, which were transferred into 24-well plates and grown in maintenance media (20% tetracycline-free FCS, 100 μg/mL hygromycin in DMEM). Clones that retained the ability to generate HBV as verified by the presence of HBs and HBV DNA in culture media were expanded and frozen in liquid nitrogen with freezing media (50% tetracycline-free FCS, 10% DMSO in DMEM).

F881 was thawed when needed and grown in maintenance media, passaged every 3 days at a ratio of 1:4. HBV production from F881 was induced by adding 250 ng/mL Doxycycline. Co-culture was performed in 12-well transwell plates (Costar) with a membrane pore size of 0.4 μm, with 8 × 10^4^ F881 cells seeded on the membrane and 1.6 × 10^5^ acceptor cells seeded in the bottom well. The co-culture shared 1.5 mL 20% tetracycline-free FCS/DMEM. Drug screen (PPI Inhibitor Library, TargetMol^®^, Boston, MA, USA) was performed at 10 μM, which is the standard concentration used for “Hit” discovery.

### 2.2. Plasmid Constructs

Overexpression constructs for HBVCP (nt1601-1860 equivalent, HBV genotype A) luciferase reporters were generated as described [21]. The insertion of 1.3 × full-length HBV replicons (nt1051-nt3221/1-nt1932 equivalent, HBV genotype A) into pcDNA3.1+ (Invitrogen, Waltham, MA, USA) upstream of the *P_CMV_* promoter through the *Mfe*I and *Mlu*I restriction sites was performed. Inducible 1.3x full-length HBV replicons were generated via insertion into pTetOne^TM^ (Takara Bio USA Inc., San Jose, CA, USA) through the *Not*I and *Mlu*I restriction sites. *SNAI2*-targeting CRISPR-Cas9 constructs were generated via the insertion of selected guide oligos into pX330 through the *Bbs*I restriction site.

### 2.3. HBV Assays

The HBV (genotype A) used to infect cells was produced from transfecting 4.5 × 10^6^ HepG2 cells with 1.3× full-length replicons [21]. To prevent carry-over plasmid contamination, transfected cells were washed vigorously thrice 24 h post-transfection with 10 mL DMEM. Precipitated HBV was stored at −80 °C as 100× stock at 3 × 10^7^ MGE/μL DMEM. A total of 1.6 × 10^5^ cells were infected in 12-well plates in the presence of 50 μM hydrocortisone, 10 μg/mL insulin and 4% PEG8000 for 24 h, then washed thrice and maintained in culture media containing 50 μM hydrocortisone and 10 μg/mL insulin.

sFISH was performed 48 h after continuous infection. Cells were washed once in PBS, fixed with 2% PFA + 20% acetic acid/PBS for 10 min at room temperature and then treated with 20% ethanol/PBS for 5 min. Probes R1 (5′ TYE^TM^ 563 GCTCTGCAGTATGGATCGGCAGAGC 3′ Iowa Black^®^ RQ Quencher) and R3 (5′ TYE^TM^ 563 GCGCTAGAAGTCCACCACGAGTCTAGCGC 3′ Iowa Black^®^ RQ Quencher) in the North2South^TM^ hybridization buffer (Thermo Fisher Scientific, Waltham, MA, USA) were incubated with treated cells on thermomixer with gentle shaking at 95 °C for 15 min and 60 °C for 15 min, then cooled to 37 °C for 1 h. Cells were counterstained at room temperature with DAPI/PBS. Fluorescence was acquired on TECAN Infinite M200.

qPCR for total HBV DNA in culture media was performed 72–96 h.p.i., as described [5,21,22], using 2 μL culture media and 5 nM primers in 20 μL reactions (LightCycler^®^480 SYBR Green I Master, Roche, Minato, Tokyo) on the LightCycler^®^480 instrument. Primers rcF (5′ TTCTTTCCCGATCATCAGTTGGACCC 3′) and rcR (5′ CCTACCTGGTTGGCTGCTGGC 3′) were used for genotype A, and rcFB (5′ TTCTTTCCCGATCACCAGTTGGACCC 3′) and rcRB (5′ CCCACCTTGTTGGAGTCCGGC 3′) were used for genotype B.

To quantitate cccDNA by “crossing the gap” [5,21,22], qPCR was performed 48 h.p.i. with 1 ng nuclear DNA digested with RNase A (Nucleospin Tissue kit, Machery-Nagel) using primers cccFA (5′ GCACCTCTCTTTACGCGGTCTCC 3′) and cccRA (5′ TGAAGCGAAGTGCACACGGACCG 3′) for genotype A, and cccFB (5′ GCACCTCTCTTTACGCGGACTCC 3′) and cccRB (5′ TGAAGCGAAGTGCAC ACGGTCCG 3′) for genotype B. Both primer pairs generate a single amplicon that melts at ~83 °C that is specific for the final ~64 bases missing from the (+) strand of rcDNA. Because deproteinated (-) strand rcDNA is robustly ligated within 1 min [23], this last stretch of (+) strand rcDNA to be completed represents the rate-limiting final step of cccDNA synthesis, in which the (+) strand of deproteinated rcDNA is completed and ligated. Because this region is the last to be completed, the cccDNA-specific primers are less likely to pick up replicative intermediates when compared to other primer sets [24] that span larger regions of rcDNA and amplify regions that are already in existence in both the (+) and (−) strands. ELISAs for HBs (Monolisa^TM^ HBs Ag Ultra, Bio-Rad, Hercules, CA, USA) and HBe (Abnova) were performed as recommended. An HBVCP luciferase reporter assay (Luciferase assay system, Promega, Tokyo, Japan) was performed as described [21].

To quantitate 3.5 kb RNAs, which comprise both pgRNA and pcRNA, qPCR was performed on 1 μL of cDNA using primers pgRNAF1 (5′ AAGGTGGGAAACTTTACGGGGCTTTATTC 3′) and pgRNAR1B (5′ GGGCAAATATTTAGTAACATTAGGATAGAACCTAG 3′). cDNA was generated from 100 ng of RNA (AccuScript High Fidelity 1st Strand cDNA Synthesis Kit, Stratagene, San Diego, CA, USA) extracted in the presence of DNase I, as recommended (Nucleospin RNA kit, Machery Nagel, Düren, Germany), to eliminate trace DNA. Expression of *GAPDH* served as loading control and was amplified using primers GAPDHF (5′ TTTGGCTACAGCAACAGGGTGGTGG 3′) and GAPDHR (5′ ATGGCAACTGTGAGGAGGGGAGATTC 3′).

### 2.4. NTCP Expression

NTCP cell surface expression was determined via flow cytometry (BD FACSCanto II) on 2 × 10^6^ live cells. The cells were washed twice, blocked in 2% BSA/PBS + 5 mM EDTA for 1 h and then stained 1:500 overnight with anti-NTCP (PA5-80001, Thermo Fisher Scientific) at 4 °C. After 3 washes, the cells were incubated with anti-rabbit AlexaFluor-488 for 1 h and washed thrice. Results were analyzed with FlowJo v10.

Endogenous full-length NTCP coding sequences (~1 kb) in NTCP transcripts were detected via RT-PCR using primers NTCPF (5′ TTAGCTAGCATGGAGGCCCACAACG 3′) and NTCPR (5′ TAACTCGAGCTAGGCTGTGCAAGGG 3′) on 3 μL of cDNA synthesized from 100 ng RNA, as described above. PCR was performed in 40 μL reactions (Expand High Fidelity PCR system, Sigma Aldrich), with cycling parameters of 95 °C for 2 min, 40 rounds of annealing at 60 °C for 30 s, elongation at 72 °C for 90 s and a final extension of 72 °C for 10 min. The amplicons generated from the primer pair were sequenced and verified to correspond to the full-length NTCP coding sequence. Expression of *GAPDH* loading control was performed as above.

When indicated, 1 μg of untagged overexpression vector for NTCP (Origene, Rockville, Maryland, USA) was transfected per 1 × 10^5^ cells 48 h before HBV infection and re-seeded at 1.6 × 10^5^ cells/well in 12-well plates. To validate the NTCP protein expression, Western blot was performed on 12 μg of membrane proteins (MEM-PER Eukaryotic Membrane Protein Extraction Reagent Kit, Thermo Fisher Scientific), using nucleoporin as the loading control. Primary antibodies (Santa-Cruz, Santa Cruz, CA, USA) used were as follows: rabbit anti-NTCP (sc-98484), rabbit anti-nucleoporin (sc-25523). Specific bands were detected using Super-Signal West Femto Maximum Sensitivity Substrate (Thermo Fisher Scientific) that was diluted 3-fold with water.

### 2.5. Immunofluorescence Staining

Staining and wash steps were performed on an orbital shaker. Cells were washed twice in PBS and then fixed in 4% PFA/PBS for 10 min. After another 2 washes with PBS, they were treated with 0.1% Triton-X/PBS for 10 min, then blocked overnight at 4 °C in blocking buffer (1% BSA/PBS). Primary antibodies were incubated at 1:200 in blocking buffer for 2 h, then washed thrice with PBS for 10 min each. Secondary antibodies (Alex Fluor, Thermo Fisher Scientific) were incubated at 1:500 in blocking buffer for 1 h in the dark. Cells were counterstained with DAPI/PBS for 10 min, then washed twice in PBS for 10 min. Primary antibodies (Santa-Cruz and Abcam) used were as follows: mouse anti-HBs (sc-23944); mouse anti-HBc (sc-23946); rabbit anti-HBx (ab39716); rabbit anti-NTCP (sc-98484); goat anti-HNF4α (sc-6556X). TUNEL assay (R&D Systems, Minneapolis, Minesotta, USA) for cytoplasmic rcDNA was performed with manufacturer’s protocol. Images were acquired using the EVOS M5000 Cell Imaging System (Invitrogen). The cccDNA fluorescence intensity for each cell was quantified by ImageJ v1.53s. 

### 2.6. Electron Microscopy

Cells were grown on collagen I (Gibco^TM^, Thermo Fisher Scientific)-coated 35 mm dishes with gridded glass coverslips (Mattek, Ashland, MA, USA), fixed in 2% PFA and 0.5% glutaraldehyde/PBS for 10 min, then stained for HBc as described above. Samples were post-fixed for 1 h with 1% buffered osmium, dehydrated in a graded series of ethanol and then infiltrated and embedded in Araldite medium. Ultrathin sections were cut using a Leica Ultracut microtome and then stained with 2% uranyl acetate and lead citrate. Observations were performed via transmission electron microscopy using an FEI Tecnai Spirit G2 at 100 kV. Images were acquired with an FEI Eagle 4K CCD camera.

### 2.7. Cell Proliferation and Cytotoxicity Assays

Cell proliferation was determined by counting the number of DAPI-stained nuclei after immunofluorescence staining. Cytotoxicity via apoptosis was determined by the presence of active executioner caspases. A Caspase-3/7 assay (Promega) was performed on F881 co-cultured on transwell membranes by transferring the membranes to empty 6-wells, and submerging cells for 1 h in the mixture of 100 μL Caspase3/7-Glo reagent and 100 μL media. Luminescence was acquired with 150 μL of the lysate transferred to clear-well black well plates.

### 2.8. Quantification and Statistical Analysis

Graphical and statistical analyses were performed using GraphPad Prism and Microsoft Excel. Student’s *t*-test was performed when appropriate to determine statistical significance. 

## 3. Results

### 3.1. HBV Acceptor Cell Lines

**Figure 1:**
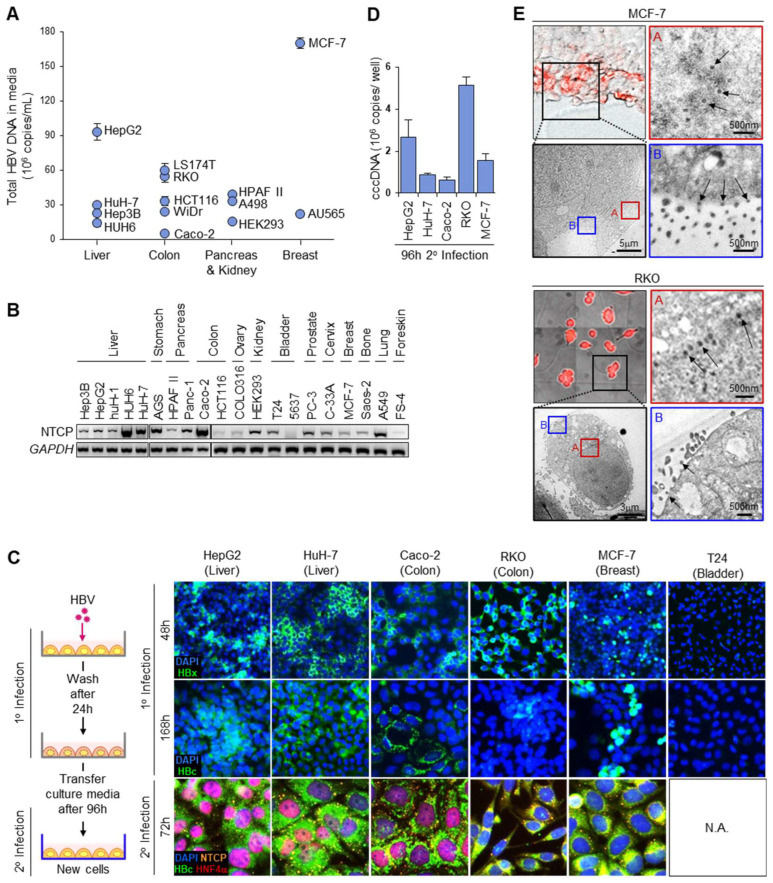
HBV acceptor cells. (**A**) Amount of HBV progeny via qPCR for total HBV DNA in culture media (n = 3, mean ± S.E.M.) from infection with HBV (genotype A). (**B**) Relative expression of NTCP full-length transcript via RT-PCR. *GAPDH* expression serves as loading control (original images can be found in supplementary). (**C**) Representative images of immunofluorescence staining (IF) for NTCP, HBx, HBc and HNF4α in HBV-infected cells, using T24 that does not support HBV replication as negative control. N.A.: Not applicable. (**D**) Nuclear cccDNA expression of HBV-infected cells via qPCR 48 h.p.i. (n = 3, mean ± S.E.M.). (**E**) Representative CLEM images (72–96 h.p.i.) staining positive for HBc (red) and actively secreting HBV (arrows). Red box A, core particles at nuclear periphery; blue box B, HBV Dane particles secreted at cell membrane.

HBV has the capacity to replicate in non-liver cells [21,25], which may be even more efficient in HBV production. To identify cells that efficiently support its entire replication cycle, 4 liver and 10 non-liver cell lines were infected with HBV (genotype A) and compared for the total HBV DNA carried by the secreted HBV progeny in the culture media (Figure 1A). The expression of HBV DNA varied extensively between cells, with the breast cancer cell MCF-7 producing the most HBV DNA at twice the levels of HepG2, which was the most efficient amongst the liver cells (HuH-7, Hep3B, HUH6) tested. Colon (LS174T, RKO, HCT116, WiDr, Caco-2), pancreas (HPAF II) and kidney (A498, HEK293) cells also actively produced HBV, with LS174T and RKO producing more HBV than HuH-7. As HBV infection cannot occur without its entry receptor NTCP (sodium taurocholate co-transporting polypeptide) [26], we determined whether this intriguing finding was associated with the surface expression of NTCP.

The live-cell surface expression of NTCP was neither negligible in non-liver cells nor significantly elevated in liver cells (Figure S1A), as non-liver cells such as A549, AGS and HCT116 stained positively for surface NTCP to a similar or greater extent than the liver cell HuH-7. The staining specificity was ascertained via overexpressed NTCP, which increased the antibody-specific fluorescence in flow cytometry (Figure S1B). Furthermore, in the Western blots for membrane proteins, the transfection of the NTCP overexpression construct into COLO316 ovary cells generated a ~55 kDa glycosylated NTCP band (Figure S1C). This band was correspondingly found in HepG2 cells, the band intensity of which increased when NTCP was overexpressed. Similar studies of non-liver cells indicate the very weak expression of NTCP by Western blot, which intensified when the in vitro-translated mRNA of NTCP was transfected [27]. The extrahepatic expression of NTCP was corroborated by the non-negligible levels of the full-length NTCP transcript in 5 liver and 15 non-liver cells (Figure 1B), indicating that the high levels of HBV production in non-liver cells, such as MCF-7, also occur through NTCP-mediated infection. The amplicons generated were sequenced, and point mutations to the NTCP coding sequence were observed for several cell lines, including HepG2 and MCF-7 (Figure S1D). However, they did not account for differences in the HBV production, as the amount of nuclear cccDNA accumulated in HUH6 liver cells overexpressing wild-type and NTCP mutants did not differ.

To confirm that HBV was actively produced from infected non-liver cells, immunofluorescence staining for de novo HBV proteins was performed (Figure 1C). HBx, an early marker of HBV replication, was strongly expressed in Caco-2, RKO and MCF-7 within 48 h.p.i., indicating that cccDNA had been established in these non-liver cells. In contrast, T24 bladder cells, which express both Slug and SOX7 transcription repressors of cccDNA [21], did not stain positive for HBx. The expression of HBc capsid protein remained evident in the HBx-positive cell types in a clonal manner with extended culture 168 h.p.i., suggesting that HBV replication remained active in them. T24 remained non-supportive for HBV replication with extended culture, as HBc expression was also negligible. To determine whether the HBV produced from non-liver cells was infectious, infected HepG2, HuH-7, Caco-2, RKO and MCF-7 were washed extensively 24 h.p.i., and then progeny HBV at 120 h.p.i. was used to infect a second batch of HBV-naïve cells via the passive transfer of the culture media (Figure 1C). The second batch of cells (2° infection) remarkably yielded homogenous staining for HBc 72 h later, which was associated with punctate cell membrane staining for NTCP. The nuclear cccDNA in them was quantified via qPCR using the established method of “crossing the gap” [5,21,22], and showed that the non-liver cells RKO and MCF-7 can support efficient HBV replication by accumulating more cccDNA at 96 h.p.i. than HuH-7 and/or HepG2 liver cells (Figure 1D). Active HBV synthesis in RKO and MCF-7 was verified via correlative light electron microscopy (CLEM), which provided direct visual evidence for HBc-positive cells containing electron-dense core particles (34 nm) at the nuclear membrane periphery and infectious Dane particles (42 nm) to be secreted at the plasma membrane (Figure 1E). Together, these data support the use of extrahepatic cells, such as MCF-7, as HBV acceptor cells in drug screen assays.

### 3.2. Maximizing Viral Production from HBV Generator Cell

**Figure 2:**
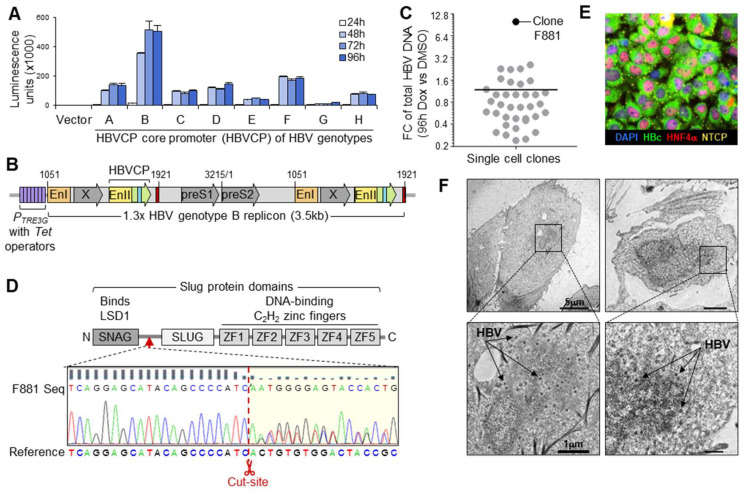
F881 HBV generator cell. (**A**) HBVCP luciferase reporter assay for HBV genotypes (A–H) in HuH-7 (n = 3, mean ± S.E.M.). (**B**) Stable transfection construct of 1.3 full-length HBV genotype B replicon in pTetOne vector for Dox-inducible HBV production. (**C**) Efficiency of single-cell clones for inducible HBV production by fold-change of total HBV DNA in culture media between Dox and DMSO treatment 96 h post-induction (n = 3, mean ± S.E.M.). (**D**) Sequence of *SNAI2* gene edits in F881 between SNAG and SLUG domains (red arrow) of Slug at intended Cas9 cut site (red scissors), removing all DNA-binding C_2_H_2_ zinc fingers. (**E**) Representative image of F881 showing all cells producing HBc (green) after 96 h Dox treatment. The cells were stained for NTCP (orange speckles on cell membrane) and nuclear HNF4α (red). (**F**) Representative TEM images of F881, showing Dane particles (42 nm, arrows) after 72 h induction.

The HBV core promoter (HBVCP) controls the transcription of pre-genomic RNA (pgRNA), which is packaged and reverse-transcribed during virus maturation to form rcDNA of infectious HBV [28]. To determine which genotype (A–H) has the most efficient HBVCP, their transcription efficiencies were compared in HuH-7 with luciferase reporter constructs (Figure 2A). Genotype B produced the highest luminescence across all time-points (24–96 h), while the other seven genotypes generated saturating luminescence at less than half the intensity. To confirm that genotype B is also the most efficient at generating other HBV components, we compared the expressions of replication markers for genotypes A–D using 1.3x full-length replicons. The replicons were inserted upstream of the *P_CMV_* promoter in the pcDNA3.1+ vector to allow HBV replication from its promoters only. Secreted surface antigen (HBs) was highest for genotype B and was confirmed via intense homogenous intracellular cytoplasmic staining (Figure S2A,B). The other genotypes secreted less HBs and produced punctate intracellular staining. Genotype B also secreted the most HBe (Figure S2C), indicating the most efficient HBVCP activity from which its 1.1x genome length pre-core RNA (pcRNA) is initiated. An inducible 1.3x full-length genotype B replicon in the pTetOne construct was thus generated (Figure 2B), allowing HBV replication to occur through HBVCP activity that was enhanced by Doxycycline acting on the contiguous *P_TRE3G_* promoter. The Tet-On system allows control over HBV production with minimal interference in downstream drug screens, as the low dose of Doxycycline used becomes negligible by the time HBV is actively produced 72–96 h later.

To remove trace amounts of HBVCP transcription repressors, HuH-7 (devoid of Sox7) was genetically edited to remove Slug, which blocks the pgRNA initiator [21]. The CRISPR-Cas9 *SNAI2*-targeting construct was co-transfected with the pTetOne replicon construct into HuH-7, together with the hygromycin selection marker, and transfectants surviving hygromycin treatment were subjected to limiting dilution in tetracycline-free media. A total of 38 single-cell clones were screened for their HBV production efficiencies by comparing the total HBV DNA content in the culture media between the DMSO and 250 ng/mL Doxycycline (Dox) treatments (Figure 2C) 96 h post-induction. Clone F881 produced the most HBV and greatest fold-change (10×). Sequencing confirmed the intended *SNAI2* knockout on both gene copies (Figure 2D). HBV was stably integrated in F881, which retained HBV production, with all cells staining intensely for HBc (Figure 2E) 96 h after the Dox treatment. This was corroborated by the TUNEL-positive cytoplasm in all the cells, as rcDNA in maturing HBV carries free 3′hydroxyl termini, which activate the TUNEL reaction (Figure S3A). Active HBV production was affirmed via TEM, which showed electron-dense Dane particles in induced F881 (Figure 2F). Infectious HBV was actively secreted by 72 h of induction, producing more HBV than the HuH-7 and HepG2 cells that had been infected through passive culture media transfer (Figure S3B). F881 continually produced HBV for 2 weeks (Figure S3C); thus, the co-cultured Dox-induced F881 continuously infected HBV acceptor cells throughout the assay period, lasting 48–72 h.

### 3.3. cccDNA Accumulation through Continuous Infection

**Figure 3:**
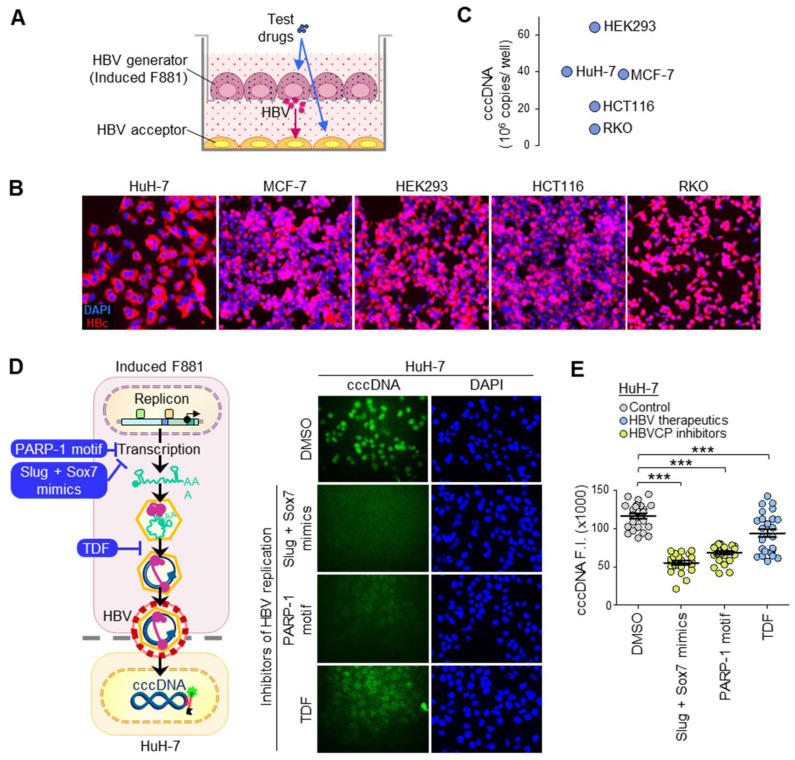
Direct fluorescence cccDNA assay. (**A**) Transwell co-culture set-up for continuous infection. (**B**) Infectious HBV from culture media of induced F881 cells at Days 4–6 after induction readily infected acceptor cells, giving homogenous HBc staining in them 96 h later. (**C**) Amount of nuclear cccDNA from continuous infection in acceptor cells at 48 h.p.i. via qPCR (n = 3, mean ± S.E.M.). (**D**) Representative images of nuclear cccDNA fluorescence in HuH-7 acceptor cells 48 h after continuous infection and concurrent treatment with 10 μM anti-HBV agents. Schematic shows how these agents reduce HBV titer in F881 generator cells to reduce cccDNA expression in acceptor cells. (**E**) Quantitation of cccDNA fluorescence from sFISH. Bars show fluorescence intensity (F.I.) (n = 25, mean ± S.E.M); *** *p* < 0.001.

To set up the fluorescent cccDNA assay, F881 cells were induced 72 h prior to seeding, and were then transferred onto a 0.4 μm transwell membrane for co-culture with acceptor cells seeded at the bottom compartment (Figure 3A). As infectious HBV is only generated ≥ 72 h.p.i., drug treatments within 48 h will only affect HBV production from F881 and affect HBV entry and cccDNA synthesis in the acceptor cells. Continuous infection enabled ~100% infection, as indicated by the homogenous HBc expression at 96 h.p.i. (Figure 3B). This generated very large amounts of cccDNA in the liver (HuH-7) and non-liver (HEK293, MCF-7, HCT116, RKO) acceptor cells to give 9-64 million copies of cccDNA per well with 48 h co-culture (Figure 3C). The >50,000-fold increase in cccDNA for HuH-7 and MCF-7 gave ~40 million copies/well (~250 copies/cell seeded), which is the upper limit found physiologically in the liver biopsies of selected individuals [29,30]. In contrast, the significantly lower levels of ~600–700 copies/well (< 1 copy/cell seeded) found in most biopsies are obtained using concentrated HBV in single-point infection models that produce clonal infection (Figure 1C). This suggests that passive media transfer attains high infectivity without the need for virus concentration or additives. Because both MCF-7 and HuH7-7 were equally efficient in HBV production, they were selected for drug screens due to their physiological relevance for liver and breast cancers [11] and their capacity to support the entire HBV life cycle.

### 3.4. Fluorescent cccDNA Assay

Molecular beacon probes have low background fluorescence due to the proximity of the 5′ TYE^TM^ 563 fluorophore and 3′ Iowa Black^®^-RQ quencher in the native stem-and-loop folded state (Figure S4A). When heated, linearized and hybridized to its target, cccDNA becomes fluorescently labeled, as the fluorophore–quencher pair are no longer in proximity. Unbound probes re-fold at room temperature, losing fluorescence, and, hence, cannot interfere with cccDNA-specific signals. cccDNA-specific probes (R1 and R3) were designed for the “gap region”, which is absent in the incomplete (+) strand of rcDNA in the cytoplasm (Figure S4B). When tested in infected HuH-7, the probes generated cccDNA-specific fluorescence in nuclei in a dose-dependent manner. In contrast, non-binding control probe R5 did not fluoresce at 1 μM (Figure S4C).

To confirm the specificity of the fluorescence signals to cccDNA, co-cultures with HuH-7 acceptor cells were treated with three different HBV replication inhibitors for 48 h to reduce the HBV production from F881 (Figure 3D), in turn reducing the cccDNA content in acceptor cells. As the clinical therapeutic [31] Tenofovir Disoproxil Fumarate (TDF) acts on the viral polymerase/reverse transcriptase (Pol/RT) and may require > 48 h to reduce the HBV titer, pre-clinical agents to suppress transcription from the HBVCP, pegylated DNA oligonucleotide PARP-1 motif (PARP-1 motif) [29] and stapled peptides mimicking Slug and Sox7 transcription repressors (Slug + Sox7 mimics) [32] were also used. All three agents significantly reduced the cccDNA-specific fluorescence intensity in HuH-7 (Figure 3E), providing confirmation that the specific loss of fluorescent cccDNA signals will yield inhibitors of HBV replication. To normalize cccDNA-specific signals, the cells were counterstained with DAPI to estimate the cell numbers and viability. Drugs targeting cccDNA specifically are distinguished from other HBV inhibitors by low ratios of cccDNA vs. DAPI signals.

### 3.5. Drug Screen for Modulators of Intranuclear cccDNA Levels

**Figure 4:**
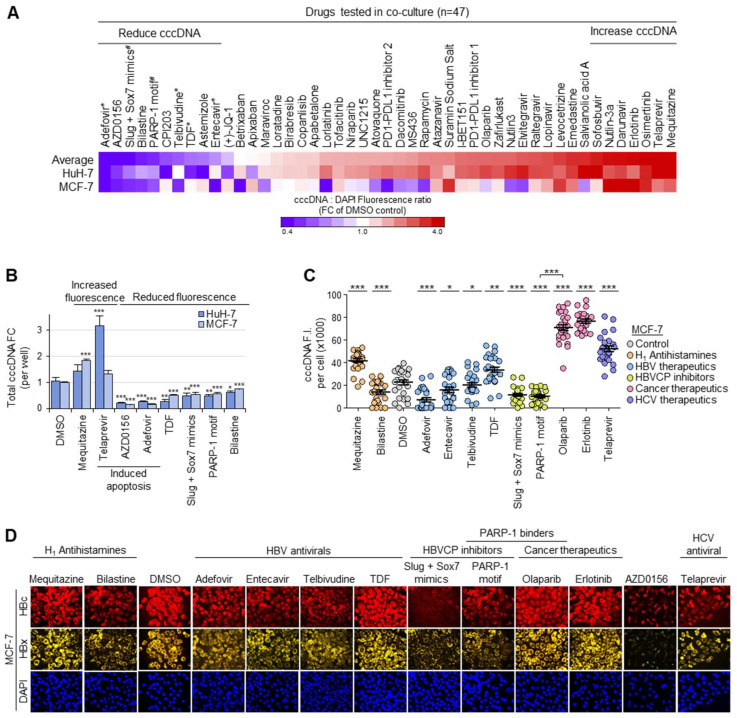
Fluorescence screen for cccDNA-targeted therapeutics. (**A**) Heatmap showing normalized cccDNA fluorescence relative to DAPI fluorescence 48 h post-drugs (n = 47, 10 μM) in HuH-7 and MCF-7 acceptor cells. Drugs that significantly increased cccDNA (mean fold-change > 3.5, n = 7) or reduced cccDNA accumulation (mean fold-change < 0.8, n = 10) relative to DMSO control in both HuH-7 and MCF-7 were selected for validation. * Clinical HBV therapeutics; ^#^ pre-clinical HBVCP inhibitors. (**B**) Effect of selected drugs via qPCR for total amount of cccDNA per well in independent experiments (n = 3, mean ± S.E.M.). *** *p* < 0.001, ** *p* < 0.01, * *p* < 0.05. (**C**) Validation of changes to normalized cccDNA fluorescence in drug screen by quantifying cccDNA-specific fluorescence in nuclei of MCF-7 48 h after drug treatment. (n = 25, mean ± S.E.M.). *** *p* < 0.001, ** *p* < 0.01, * *p* < 0.05. (**D**) Representative images corresponding to protein expression of HBc (red) and HBx (yellow) 48 h post-drug-treatment in MCF-7.

The drug screen platform was tested using a panel of drugs (n = 47), consisting of four clinical HBV therapeutics [31] (Adefovir, Telbivudine, TDF, Entecavir), two combinations of small molecules against HBVCP activity (PARP-1 motif, Slug + Sox7 mimics) as well as other FDA-approved clinical or trial drugs that disrupt protein–protein interactions (Figure 4A). It was encouraging that all HBV therapeutics and inhibitors of HBVCP activity were correctly identified to give significant losses in normalized cccDNA fluorescence relative to the DMSO control, indicating that the cells from these treatments had significantly lower cccDNA levels per cell than the DMSO control. Drugs that reduced the average normalized fluorescence by > 20% included AZD0156 (DNA checkpoint protein ATM inhibitor), CP1203 (inhibitor of BET bromodomain found in epigenetic regulators) as well as the antihistamines Bilastine and Astemizole. Interestingly, many drugs, including the anticholinergic H_1_ antihistamine Mequitazine, HCV antiviral Telaprevir and EGFR (Epithelial Growth Factor Receptor) inhibitors Osimertinib and Erlotinib, increased the normalized cccDNA fluorescence, indicating that they induced HBV replication, HBV entry or cccDNA synthesis.

### 3.6. Functional Validation of cccDNA-Targeted Drug Screen

To validate the drug screen, co-cultured F881 cells were lysed and analyzed (Figure S5A) for cytotoxicity via an apoptosis assay. Many of them, including the HBV therapeutic Adefovir and the antineoplastic antihistamine Astemizole, induced apoptosis (Figure S5B). Non-toxic agents that reduced cccDNA in both HuH-7 and MCF-7 included the remaining three HBV therapeutics, HBVCP inhibitors and the novel modulator Bilastine, which relieves allergy. Amongst them, only the HBVCP inhibitors and Bilastine did not affect the cell proliferation of HBV acceptor cells, producing no difference in the cell counts when compared to the DMSO control after 48 h of drug treatment (Figure S5C).

To ascertain that the changes in the normalized cccDNA fluorescence in the viable cells corresponded to the net loss of cccDNA in the culture, an independent co-culture was set up, and the total amount of cccDNA per well was determined via qPCR. The qPCR data concur with the normalized cccDNA fluorescence data, such that cytotoxic AZD1056 and Adefovir consistently yielded the lowest cccDNA levels in both HuH-7 and MCF-7 (Figure 4B). Similarly, increased normalized fluorescent cccDNA signals from Mequitazine and Telaprevir were verified by the increased cccDNA levels. The HBVCP inhibitors also correspondingly reduced the cccDNA in MCF-7 and HuH-7 to almost half that of the DMSO control. The assay accurately identified TDF as having differential effects between HuH-7 and MCF-7, as the surprisingly minimal effect on the normalized cccDNA levels in the viable MCF-7 cells was reflected by the lesser loss in the total cccDNA content for MCF-7 when compared to HuH-7. Because the altered cccDNA expression in acceptor cells may have been brought about by corresponding changes in the HBV titer from F881, its specificity on cccDNA was ascertained by observing no loss in the total HBV DNA content in the culture media when compared to the DMSO control (Figure S5D). In contrast, the HBVCP inhibitors reduced the HBV production from F881; thus, their low normalized cccDNA fluorescence corresponded to the anticipated loss of HBV DNA content in the culture media. Similarly, the corroborated increase in the normalized cccDNA fluorescence and HBV DNA content in the culture media for the Telaprevir and Mequitazine treatments indicate that they enhanced the HBV production from F881 and do not directly target cccDNA. Together, these results validate the accuracy of the cccDNA fluorescent assay; hence, the significant loss of cccDNA in both HuH-7 and MCF-7 with the Bilastine treatment is not an artefact.

To functionally validate the changes in the cccDNA levels, the fluorescence intensity of the cccDNA was corroborated by the corresponding fluorescence intensities for HBx and HBc. DMSO-treated cells showed cccDNA-specific fluorescence in the nuclei of MCF-7 (Figure S5E), and the cccDNA-specific fluorescence intensity of each nucleus (Figure 4C) corresponded to the cytoplasmic staining for HBx and HBc (Figure 4D). Consistent with the drug screen results, Mequitazine-, Telaprevir- and Erlotinib-treated cells stained more intensely for cccDNA, HBx and HBc. Furthermore, the fluorescence intensity for the cccDNA corresponded to the normalized cccDNA:DAPI fluorescence ratios in MCF-7 (Figure 4A), where Adefovir, with the weakest staining, produced the lowest ratio, followed by Entecavir and Telbivudine with intermediate intensities of staining and ratios, and TDF with the strongest cccDNA-specific staining intensity, similar to the DMSO control, producing a negligible change in the ratio when compared to the DMSO control. In further support of the fluorescence cccDNA assay, both HBVCP inhibitors significantly reduced the cccDNA staining to barely detectable levels, with correspondingly weak staining for HBx and HBc. Of note, the pegylated PARP-1 motif is a PARP-1-specific antagonist that blocks transcription at the HBVCP [32], reducing the cccDNA, HBx and HBc fluorescence intensities. In stark contrast, the clinical PARP inhibitor Olaparib facilitates PARP-1-dependent transcription at the HBVCP by reducing PARP-1 auto-ribosylation [33], producing the opposite effect of enhancing the cccDNA, HBx and HBc signals. The novel cccDNA-targeting agent Bilastine also displayed a loss of staining for cccDNA, HBc and HBx. Cytotoxic AZD0156 ablated the cccDNA fluorescence and produced the least HBx and HBc, accurately reflecting the significant loss in the normalized cccDNA fluorescence. Thus, the drug screen platform enables the identification of cccDNA-directed HBV therapeutics in a high-throughput manner, which was simultaneously functionally verified by multiplexed immunofluorescence staining for HBx and HBc, and further supported by cytotoxicity data from apoptosis assays performed on F881 in a single set-up (Figure S5A).

### 3.7. Structurally Similar Antihistamines Downregulate cccDNA

**Figure 5:**
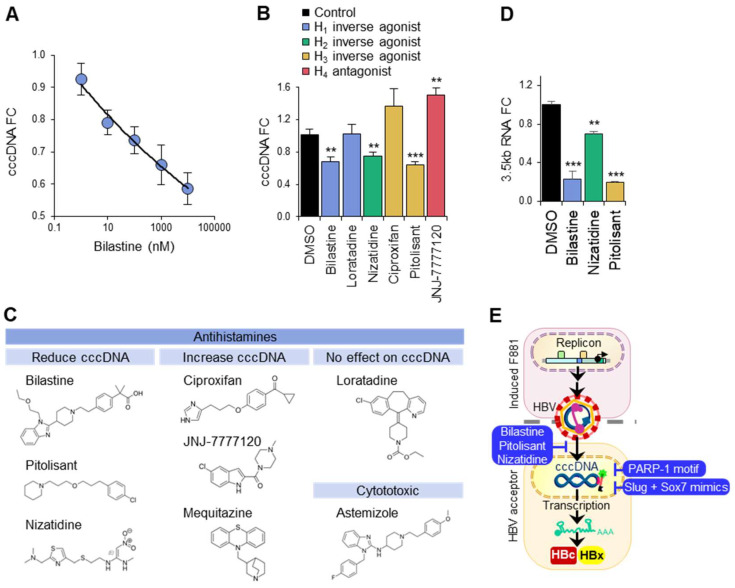
cccDNA downregulation via antihistamines. (**A**) Dose-dependent effect of Bilastine on nuclear cccDNA in HuH-7 acceptor cells. (**B**) Relative expression of cccDNA via qPCR in HuH-7 acceptor cells treated with 10 μM antihistamines. *** *p* < 0.001, ** *p* < 0.01. (**C**) Chemical structures of antihistamines and their effect on cccDNA. (**D**) Effect of antihistamines on 3.5 kb RNAs (pgRNA and pcRNA) in HuH-7 acceptor cells via qPCR. *** *p* < 0.001, ** *p* < 0.01. (**E**) Suppressors of cccDNA levels identified through fluorescence cccDNA-targeted drug screen.

Because Bilastine was identified to downregulate cccDNA in HuH-7 liver cells in a dose-dependent manner (Figure 5A), we determined whether other H_1_ antihistamines (Loratadine) and antihistamines for H_2_, H_3_ and H_4_ histamine receptors (Nizatidine, Ciproxifan, Pitolisant, JNJ-7777120) had a similar effect. Surprisingly, Loratadine did not affect the cccDNA levels in HuH-7 acceptor cells co-cultured for 48 h with F881. Instead, the H_2_ and H_3_ antihistamines Nizatidine and Pitolisant, respectively, downregulated the cccDNA to a similar extent as Bilastine. In stark contrast, the H_3_ and H_4_ antihistamines Ciproxifan and JNJ7777120, respectively, upregulated the cccDNA significantly (Figure 5B). It appears that the antihistamines that exerted significant effects on cccDNA did so independent of their target histamine receptor sub-type and possess structures (Figure 5C) that differ from that of Loratadine. The loss of cccDNA associated with Bilastine, Pitolisant and Nizatidine was functionally validated in HuH-7 acceptor cells via qPCR for 3.5 kb RNAs (pgRNA and pcRNA), which were drastically reduced by up to 70% for the three suppressors of cccDNA levels (Figure 5D). Thus, our cccDNA-targeted drug screen successfully identified Bilastine and a group of structurally similar antihistamines (Figure 5E) as suppressors of cccDNA levels that act upstream to block cccDNA formation.

## 4. Discussion

The persistence of cccDNA is associated with delayed viral clearance, chronic infection and HBV reactivation, but this problem is not directly addressed by current therapeutics, as they cannot eliminate cccDNA. To address the lack of cccDNA-targeted therapeutics, we developed a platform to massively amplify cccDNA copies in vitro, facilitating detection and quantification through fluorescent labels for the purpose of high-throughput drug screens (Figure 4A). This was achieved through continuous co-culture, in which the proximity between the HBV donor and HBV acceptor cells, together with the active, ongoing secretion of large amounts of fresh, viable and unaltered HBV, enabled the high infectivity of HBV acceptor cells. Other active processes, such as the uptake of exosomes containing HBV and its nucleic acids from donor cells into acceptor cells [34], may also have contributed to the very high amount of cccDNA observed. The loss of the cccDNA:DAPI fluorescence ratio with Adefovir in both HuH-7 and MCF-7 demonstrates the robustness and sensitivity of the cccDNA assay, as slight changes in cccDNA expression that take time to manifest in vivo are accelerated in our drug screen system with massively amplified cccDNA. The low cccDNA:DAPI fluorescence ratio concurred with the significant loss in the total cccDNA titer in both HuH-7 and MCF-7, which corresponded to the loss in the cccDNA-specific fluorescence intensity in the cell nuclei. Furthermore, the non-destructive nature of the cccDNA assay allows the functional validation of the cccDNA levels via coupling with immunofluorescence staining (Figure 4D), which correlates well with the cccDNA fluorescence intensity of each cell. 

Using a small FDA-approved panel, we identified the H_1_ antihistamine Bilastine as a therapeutic already widely used for allergies [35] to suppress liver and non-liver cccDNA levels without cytotoxicity. In contrast, current clinical therapeutics, such as TDF, Telbivudine and Entecavir, cannot consistently reduce the cccDNA, HBx and HBc levels within 48 h. This is consistent with the need for their long-term administration to clinically reduce HBV titers. While the exact mechanism of Bilastine in suppressing cccDNA levels remains to be elucidated, it has been reported that H_1_ antihistamines reduce the incidence of HBV-associated liver cancer [36] by ~50%. Indeed, cccDNA levels directly correlate with the risk of developing liver cancer and tumor recurrence [37,38], in part by reducing the infected cell’s capacity for DNA repair through the sequestration of the DNA repair enzyme PARP-1 [32]. Thus, the finding that Bilastine suppresses cccDNA levels may be clinically relevant, prompting further investigation into its incorporation for HBV treatment regimes.

Interestingly, H_1_ antihistamines are not functionally equivalent to Bilastine, as Mequitazine cannot reduce the cccDNA levels in HuH-7 and consistently increased the HBV replication in MCF-7 (Figure 4A–C). “T-shaped” Mequitazine, like another H_1_ antihistamine, Methapyrilene hydrochloride, which upregulates DNA repair synthesis by 7-fold [39], may enhance cccDNA synthesis by increasing the efficiency of DNA repair. Even though Loratadine shares this structure, it has a more complex “stalk”, which may account for its lack of effects on cccDNA in HuH-7 (Figure 5B). Astemizole, which is structurally more complex, is, in contrast, cytotoxic (Figure S5B). Amongst the antihistamines tested, only the H_2_ and H_3_ antihistamines Nizatidine and Pitolisant, respectively, downregulated the cccDNA to a similar extent as Bilastine (Figure 5C). Several antihistamines inhibit viral infection through viral-receptor-specific and non-specific mechanisms, including the H_1_ antagonists Carbinoxamine Maleate and Chlorpheniramine Maleate against influenza viruses [40], the H_1_ antagonist Chlorcyclizine against hepatitis C virus [41] and the H_2_ antagonist Famotidine against SARS-CoV-2 [42]. As Nizatidine is similar in structure to Famotidine, being a curved 1,3-thiazole, it may behave like Famotidine to block HBV entry in a histamine-dependent manner. Surprisingly, Bilastine and Pitolisant were more potent than Nizatidine in reducing 3.5 kb transcripts (Figure 5D), suggesting that these long molecules bridging aromatic moieties in angled planes may share additional functions in suppressing cccDNA transcription. Intriguingly, Curcumin, a natural compound with a similar structure known for blocking the histamine release from mast cells [43], has been predicted to be a potent inverse H_1_ agonist [44]. More importantly, it suppresses cccDNA transcription by downregulating histone acetylation [45] and binds NTCP directly to block HBV uptake [46]. These findings suggest that Bilastine and Pitolisant likely possess dual HBV suppressive activities: HBV infection blockade to reduce the cccDNA titer, and cccDNA inactivation to reduce HBV transcripts. Taken together, the cccDNA-suppressive effects of antihistamines are unlikely to be directly mediated through histamine receptor signaling and may be brought about by multiple mechanisms that block HBV entry, cccDNA synthesis and cccDNA activity in a structure-dependent manner.

Besides direct modulators of the cccDNA levels, agents suppressing the cccDNA function were also identified. The HBVCP inhibitors reduced the cccDNA levels and suppressed the cccDNA function in both HuH-7 and MCF-7 without increasing HBV production, suggesting that they may be developed as novel HBV therapeutics. In particular, the pegylated PARP-1 motif may be relevant for HBV-associated liver cancer, as the PARP-1-specific antagonist inhibits both PARP-1-dependent DNA repair [32] and PARP-1-dependent transcription at the HBVCP, sensitizing liver cancer cells to cell death from irreparable damage and reducing the HBV production from F881 (Figure S5C) to suppress the cccDNA levels in acceptor cells (Figure 3D,E and Figure 4C). In contrast, the clinical PARP inhibitor Olaparib [47] enhanced the cccDNA fluorescence (Figure 4C) and increased the expression of HBx and HBc (Figure 4D) by preventing PARP-1 auto-ribosylation, which enhances its retention on cccDNA [33]. With increasing public acceptance for nucleic acid therapeutics, the dual effect of the pegylated PARP-1 motif makes it a particularly appealing agent for treating HBV-associated cancers.

The drug screen would also be useful for identifying therapeutics that enhance HBV replication in its target patients. The EGFR inhibitors Erlotinib and Osimertinib were, surprisingly, found to upregulate cccDNA (Figure 4A,C,D). EGFR is NTCP’s co-receptor, which internalizes the HBV-NTCP-EGFR complex such that it is co-localized intracellularly upon infection [48,49]. Because EGFR inhibitors trigger EGFR internalization [50,51], perhaps the increased internalization of the HBV-NTCP-EGFR complex resulted in the increase in the cccDNA and HBV replication in the HBV acceptor cells. This phenomenon is physiologically supported by clinical reports on HBV reactivation in patients treated with EGFR inhibitors, in which 9.63% of patients displayed a 10-fold increase or >10^5^ IU/mL of HBV DNA [52]. The HCV antivirals Telaprevir and Sofosbuvir were also identified to enhance HBV replication, which is supported by FDA-issued warnings of HBV reactivation [53].

To validate the mechanism of HBV infection in the non-liver cells tested, we found that many of them expressed full-length transcripts of NTCP (Figure 1B). This was affirmed by the protein expression at the cell surface through immunofluorescence staining (Figure 1C), flow cytometry (Figure S1A) and Western blot for membrane proteins (Figure S1C). Our data concur with the #HPA042727 dataset from The Human Protein Atlas, which, via immunohistochemistry, showed that NTCP was expressed in liver hepatocytes, respiratory epithelial cells and multiple types of glandular cells, including those from the kidney tubules, stomach, pancreas, prostate and breast [54]. Moreover, NTCP expression was found in cancers of such tissues. Interestingly, NTCP often has single-residue mutations in these cell lines, including the HepG2 and Hep3B liver cell lines (Figure S1D), likely affecting its detection in multiple reports. The common I223T and L137R mutations in HepG2 and MCF-7 did not affect the cccDNA accumulation, and these cells remained highly efficient in HBV infection and production (Figure 1). As the trace expression of NTCP on the cell surface is sufficient for HBV infection in non-liver cells, such as MCF-7 (Figure 1B,C and Figure S1A), and because the relative expression of NTCP expression does not correlate with the relative expression of the cccDNA and HBV DNA content in culture media, NTCP is not the only determinant for efficient HBV infection [55,56,57].

Indeed, several non-liver cells, such as MCF-7, support the entire HBV replication cycle to produce infectious HBV efficiently (Figure 1A,E). It has been reported that NTCP is upregulated in breast cancer tissues, which were found via electron microscopy to contain HBV in HBV-positive patients [11]. When interpreted with the mounting evidence of extrahepatic HBV replication and the association of HBV with extrahepatic diseases and cancers [11,12,13,14,15,16], drug screens to identify curative therapies that are also efficacious for hidden HBV pools should encompass a range of HBV-permissive cells, such as MCF-7. This would address potential cell-type-specific differences in the HBV replication mechanisms, such as the lack of multiple nuclear HNF4α isoforms in MCF-7 (Figure 1C), that may aid drug resistance to enable HBV persistence in the extrahepatic pools. Differences between the cccDNA:DAPI fluorescence ratio between HuH-7 and MCF-7 were observed for several drugs, including the HBV antivirals Entecavir, TDF and Telbivudine, suggesting that antivirals efficacious in the liver need not be efficacious in non-liver reservoirs of HBV. By using both liver (HuH-7) and non-liver (MCF-7) cells in the drug screen (Figure 4A–C), “hits” identified through our drug screen would very likely be efficacious in both the liver and extrahepatic tissues, providing higher confidence for obtaining curative therapeutics aimed at stamping out HBV.

## 5. Conclusions

In conclusion, our cccDNA-targeted drug screen platform allows for cccDNA amplification in acceptor cells to enable the rapid screening of drug libraries with high accuracy. As the fluorescence cccDNA assay is non-destructive, the functional loss of cccDNA is simultaneously validated via the concurrent staining for HBx and HBc. False positives that have little effect on the cccDNA amount per cell are directly identified through the negligible loss of cccDNA-specific fluorescence relative to nuclear staining with DAPI. The H_1_ antihistamine Bilastine was identified to safely lower cccDNA levels in liver and non-liver cells, and HBVCP inhibitors were verified as novel therapeutics to suppress hepatic and extrahepatic HBV replication by rapidly downregulating de novo cccDNA synthesis. The pegylated PARP-1 motif may also be relevant for HBV-associated cancers, as it also sensitizes cancer cells to DNA-damaging agents often used in cancer therapy. By adapting the methodology of continuous infection followed by sFISH, the platform may be pivoted for the identification of viral genome-directed therapeutics against other persistent viruses.

## Data Availability

No new data created.

## Supplementary Materials

The following supporting information can be downloaded at https://www.mdpi.com/article/10.3390/biom13101438/s1, Figure S1: Expression of functional NTCP on cell membrane of non-liver cells; Figure S2: HBV genotype B replicates efficiently in HuH-7; Figure S3: HBV generator cell F881 produces infectious HBV; Figure S4: Fluorescent labeling of nuclear cccDNA via simplified FISH (sFISH) using molecular beacon probes; Figure S5: Simultaneous functional validation of cccDNA drug screen.

## Abbreviations

cccDNA	Covalently closed circular DNA
CLEM	Correlative light and electron microscopy
Dox	Doxycycline
HBV	Hepatitis B virus
HBVCP	HBV core promoter
HNF4α	Hepatocyte nuclear factor 4α
h.p.i.	Hours post-infection
MGE	Multiplicities of genome equivalents
Pol/RT	Polymerase/reverse transcriptase
pcRNA	pre-core RNA
pgRNA	pre-genomic RNA
rcDNA	Relaxed closed circular DNA
sFISH	Simplified fluorescence in situ hybridization
TEM	Transmission electron microscope
