# Supplementary material for: cccDNA-Targeted Drug Screen Reveals a Class of Antihistamines as Suppressors of HBV Genome Levels
Source: Biomolecules. 2023 Sep 24;13(10):1438. doi: 10.3390/biom13101438 (PMC10604930; doi:10.3390/biom13101438)
Supplement: Supplementary file 1 [file biomolecules-13-01438-s001.zip › biomolecules-2541369-original-images.pdf]

Original Gel Images for Figure 1B

Original gel for transcript of full-length NTCP (Coding sequence = 1047bp)

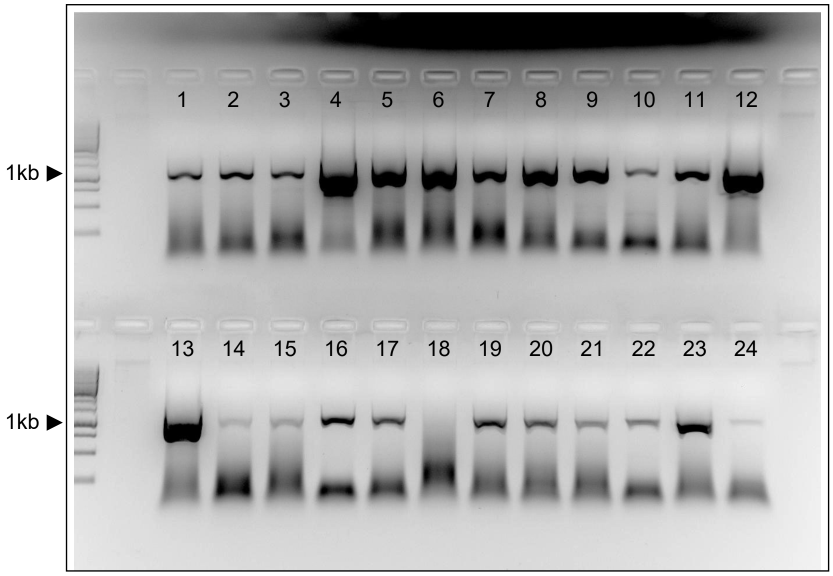

1. Hep3B
2. HepG2
3. huH-1
4. HUH6
5. HuH-7
6. Primary human hepatocyte #1*
7. Primary human hepatocyte #2*
8. Primary human hepatocyte #3*
9. AGS
10. HPAF II
11. Panc-1
12. Caco-2
13. Caco-2*
14. HCT116
15. COLO316
16. HEK293
17. T24
18. 5637
19. PC-3
20. C-33A
21. MCF-7
22. Saos-2
23. A549
24. FS-4

*Lanes omitted in Figure 1B

Original gel for 3’ end of *GAPDH* transcript (Specific band = 178bp)

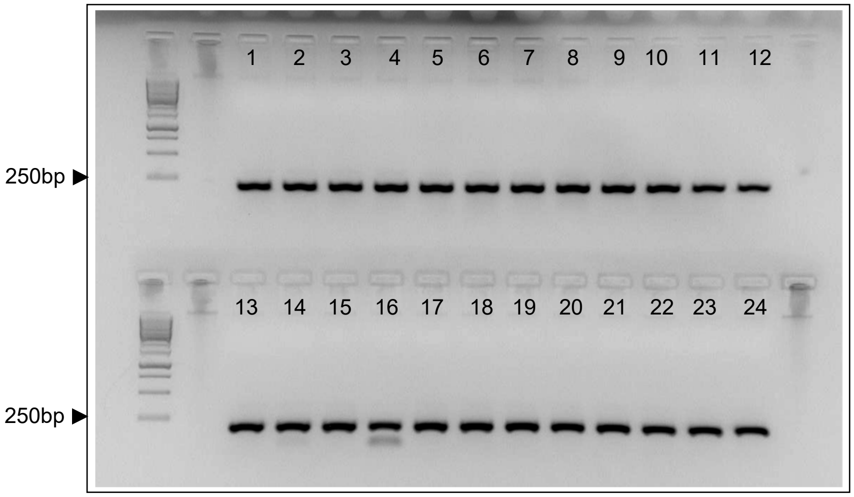

1. Hep3B
2. HepG2
3. huH-1
4. HUH6
5. HuH-7
6. Primary human hepatocyte #1*
7. Primary human hepatocyte #2*
8. Primary human hepatocyte #3*
9. AGS
10. HPAF II
11. Panc-1
12. Caco-2
13. Caco-2*
14. HCT116
15. COLO316
16. HEK293
17. T24
18. 5637
19. PC-3
20. C-33A
21. MCF-7
22. Saos-2
23. A549
24. FS-4

*Lanes omitted in Figure 1B

Original Blot for Figure S1C

Original blot for NTCP and Nucleoporin

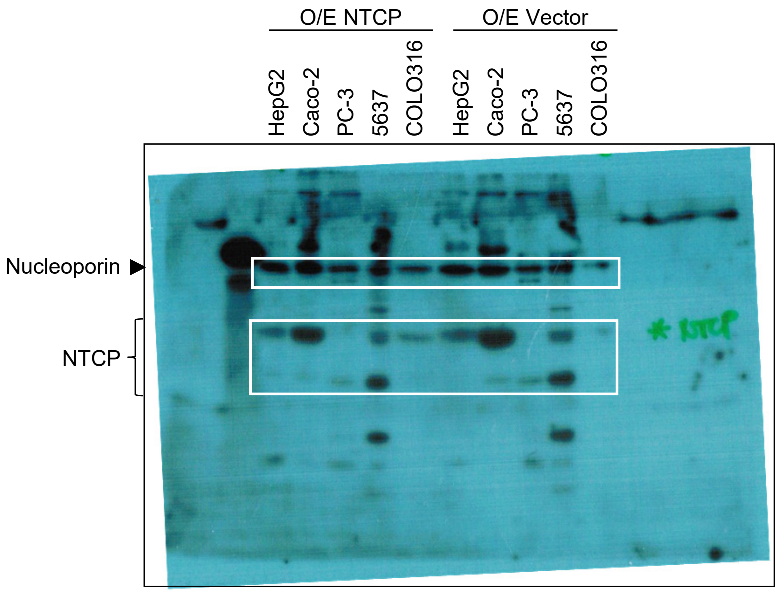

Boxed areas show cropped image for Figure S1C.
