# Supplementary material for: cccDNA-Targeted Drug Screen Reveals a Class of Antihistamines as Suppressors of HBV Genome Levels
Source: Biomolecules. 2023 Sep 24;13(10):1438. doi: 10.3390/biom13101438 (PMC10604930; doi:10.3390/biom13101438)
Supplement: Supplementary file 1 [file biomolecules-13-01438-s001.zip › biomolecules-2541369-supplementary.pdf]

## Supplementary Figures

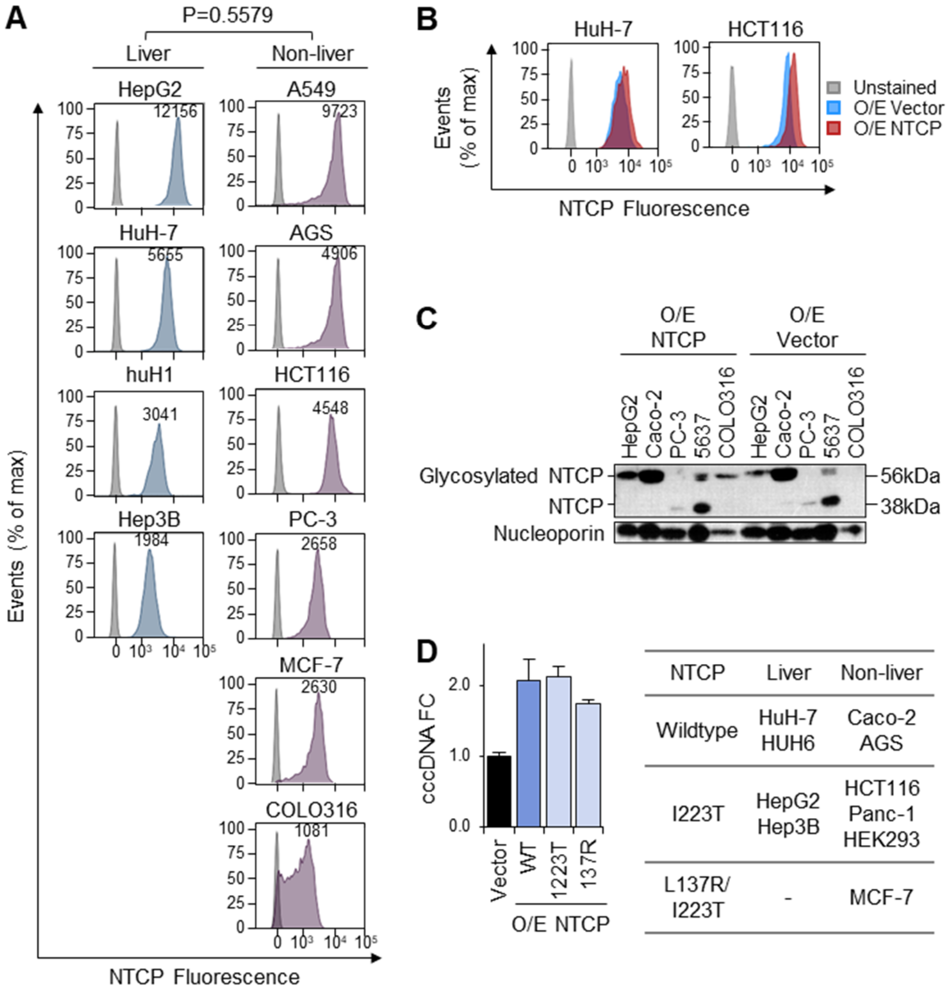


**Figure S1.** Expression of functional NTCP on cell membrane of non-liver cells. (**A**) Cell surface expression of NTCP by flow cytometry. Mean fluorescence intensity of cells stained with $\alpha$-NTCP is indicated by numbers on peaks. Control peaks are grey. (**B**) Specificity of NTCP staining in flow cytometry is demonstrated by increased mean fluorescence intensity upon overexpression of wildtype NTCP. (**C**) NTCP is expressed in the membrane fraction of non-liver cells. Antibody specificity is demonstrated by the band when wildtype NTCP is overexpressed (O/E) in COLO316, which is absent in COLO316 transfected with empty vector. The corresponding band in control HepG2 is also more intense when NTCP is specifically overexpressed. Nucleoporin was used

as loading control. (**D**) Mutations in NTCP coding sequence from cell lines by Sanger sequencing. Relative to wildtype control, overexpressed NTCP mutants do not affect increased accumulation of cccDNA in HUH6, indicating no loss in HBV infection.

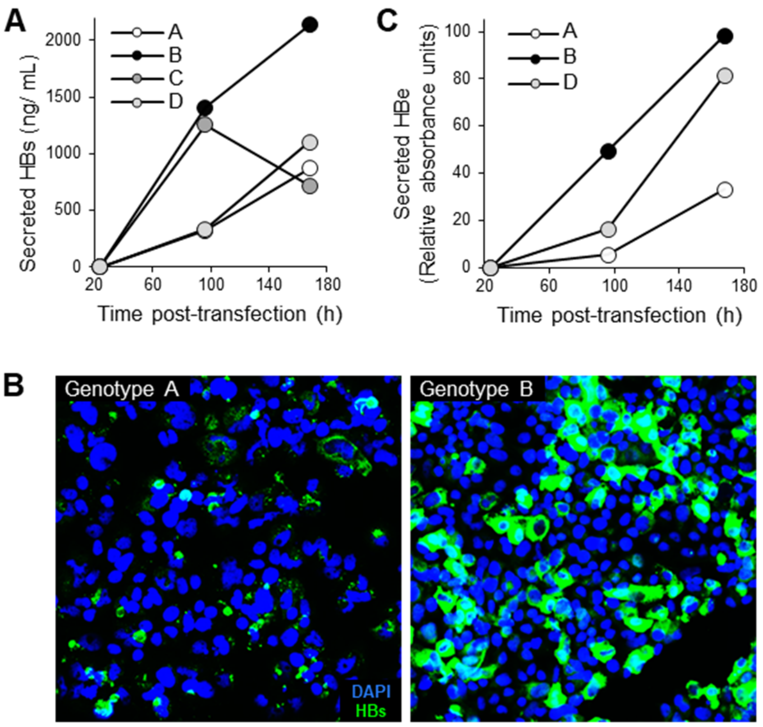


**Figure S2.** HBV genotype B replicates efficiently in HuH-7. HuH-7 was transfected by 1.3x full-length replicon constructs and tested for HBV replication markers. (**A**) HBs secretion by ELISA (n=3, mean±S.E.M.) (**B**) Typical immunofluorescence staining for intracellular HBs. (**C**) HBe secretion by ELISA (n=3, mean±S.E.M.).

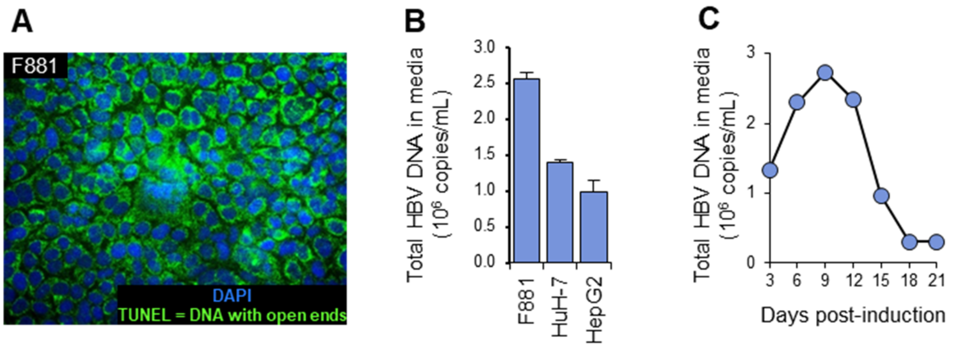


**Figure S3.** HBV generator cell F881 produces infectious HBV. (**A**) F881 cells are TUNEL-positive in the cytoplasm, as rcDNA in them is partially double-stranded with open 3' ends that are TUNEL reactive. (**B**) HBV generated from F881 is infectious, as passive transfer of culture media from Days 4-6 of induced F881 successfully infected HuH-7 and HepG2 acceptor cells 72h later to generate progeny HBV. (**C**) HBV production in F881 was sustained >2 weeks by passaging cells in the ratio of 1:4 every 3 days with addition of fresh Dox per passage.

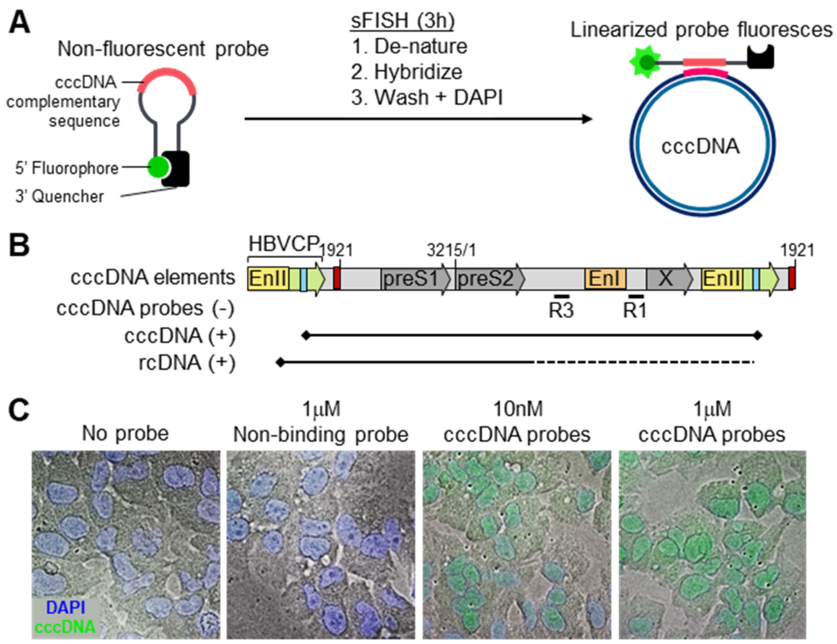


**Figure S4.** Fluorescent labelling of nuclear cccDNA by simplified FISH (sFISH) using molecular beacon probes. (**A**) sFISH molecular beacon probe design for cccDNA and staining procedure. (**B**) cccDNA-specific probes R1 and R3 specifically hybridize to the complete (+) strand of cccDNA, which is absent in rcDNA of infectious virions. (**C**) Representative images showing nuclear cccDNA-specific fluorescence (green) from sFISH of infected HuH-7. Non-specific probe R5 serves as negative control.

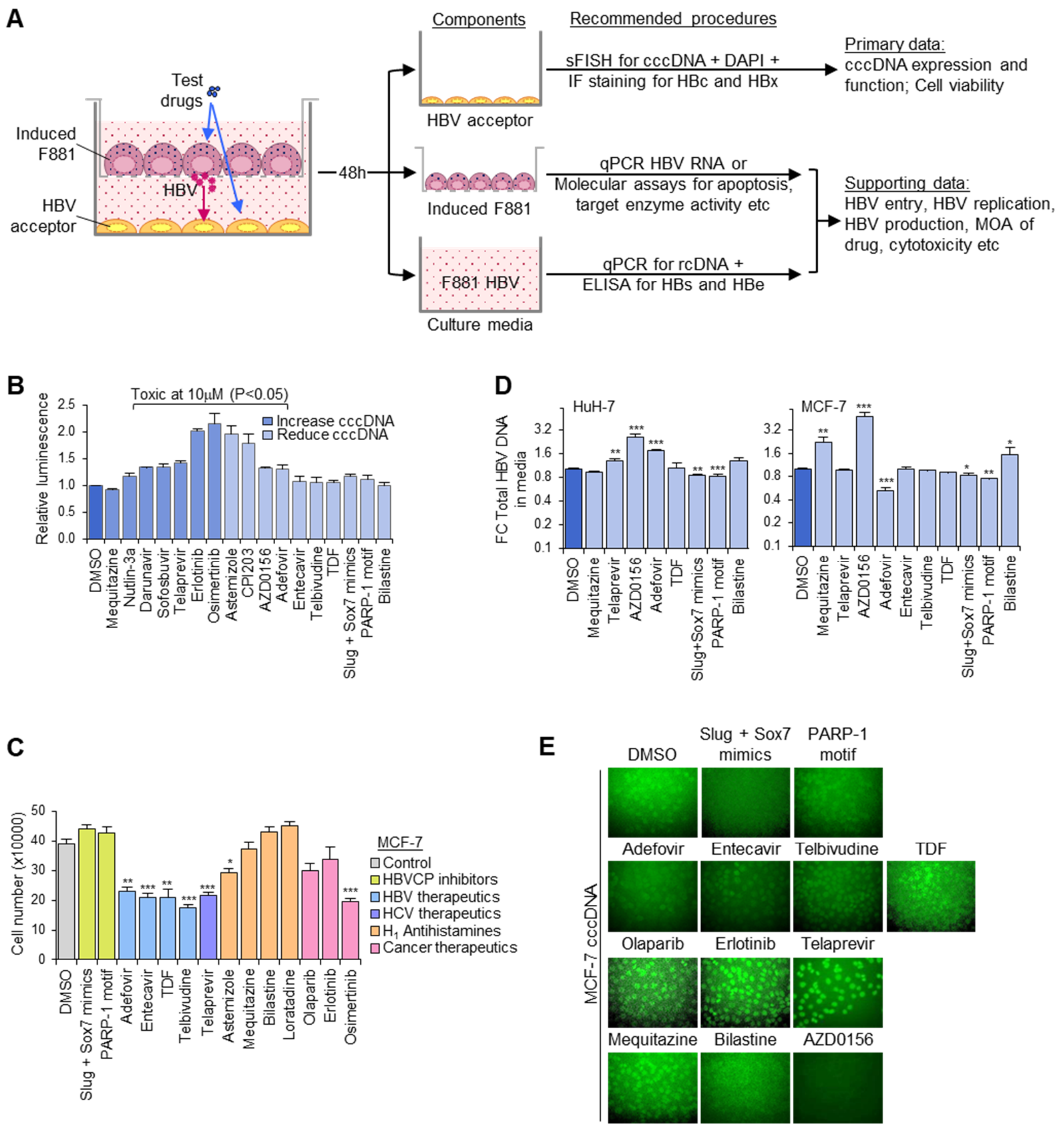


**Figure S5.** Simultaneous functional validation of cccDNA drug screen. (**A**) Besides using HBV acceptor cells for the fluorescent cccDNA assay, the schematic shows how other components of the co-culture can be used to generate additional information about cell viability and HBV replication. MOA: mechanism of action. (**B**) Apoptosis assay for caspase-3/7 (n=3, mean±S.E.M.) in co-cultured F881 cells treated with drugs

for 48h. $p<0.05$ for indicated drugs. (**C**) MCF-7 HBV acceptor cell count 48h post drug treatment. (n=3, mean±S.E.M.). *$p<0.05$, **$p<0.01$, ***$p<0.001$. As all samples yielded more than 16 $x10^4$ cells per well, which is the original number of cells seeded, none of the drug treatments tested at industry standards of 10μM was cytotoxic for MCF-7. (**D**) qPCR of total HBV DNA content from infectious HBV generated from drug treated F881 (n=3, mean±S.E.M.). *$p<0.05$, **$p<0.01$, ***$p<0.001$. (**E**) Representative images for fluorescent nuclear cccDNA in acceptor cells treated with 10μM of indicated drugs in MCF-7.
